# Relationships between Dietary Patterns and Erythropoiesis-Associated Micronutrient Deficiencies (Iron, Folate, and Vitamin B_12_) among Pregnant Women in Taiwan

**DOI:** 10.3390/nu15102311

**Published:** 2023-05-15

**Authors:** Noor Rohmah Mayasari, Chyi-Huey Bai, Jane C.-J. Chao, Yi-Chun Chen, Ya-Li Huang, Fan-Fen Wang, Bayu Satria Wiratama, Jung-Su Chang

**Affiliations:** 1 School of Nutrition and Health Sciences, College of Nutrition, Taipei Medical University, Taipei 11031, Taiwan or noormayasari@unesa.ac.id (N.R.M.);; 2 Department of Nutrition, Faculty of Sports and Health Sciences, Universitas Negeri Surabaya, Surabaya 60213, Indonesia; 3 School of Public Health, College of Public Health, Taipei Medical University, Taipei 11031, Taiwan; 4 Department of Public Health, School of Medicine, College of Medicine, Taipei Medical University, Taipei 11031, Taiwan; 5 Department of Internal Medicine, Yangming Branch, Taipei City Hospital, Taipei 11146, Taiwan; 6 Department of Biostatistics and Epidemiology, Faculty of Medicine, Public Health and Nursing, Universitas Gadjah Mada, Yogyakarta 55281, Indonesia; 7 Graduate Institute of Metabolism and Obesity Sciences, College of Nutrition, Taipei Medical University, Taipei 11031, Taiwan; 8 Nutrition Research Center, Taipei Medical University Hospital, Taipei 11031, Taiwan; 9 Chinese Taipei Society for the Study of Obesity (CTSSO), Taipei 100, Taiwan; 10 TMU Research Center for Digestive Medicine, Taipei Medical University, Taipei 11031, Taiwan

**Keywords:** dietary pattern, erythropoiesis-associated micronutrient deficiencies, pregnant women, household income, anemia

## Abstract

Globally, anemia affects 56 million pregnant women, especially women with a low household income. Functional erythropoiesis requires a constant supply of micronutrients, and the demands significantly increase during fetal development. This study aims to identify dietary patterns for preventing gestational erythropoiesis-associated micronutrient deficiencies (e.g., iron, folic acid, and vitamin B_12_). A Nationwide Nutrition and Health Survey in Pregnant Women, Taiwan (NAHSIT-PW), was conducted between 2017 and 2019. Data on baseline information, diet, anthropometrics, and blood biochemistry were collected during a prenatal visit. Dietary patterns were identified using a reduced rank regression (RRR). Erythropoiesis-related micronutrient deficiencies were defined as single, double, and triple micronutrient deficiencies of an iron deficiency, folate depletion, and a vitamin B_12_ deficiency. In total, 1437 singleton pregnancies aged ≥20–48 years were included in the analysis. Prevalences of normal nutrition, and single, double, and triple erythropoiesis-related micronutrient deficiencies were 35.7%, 38.2%, 18.6%, and 7.5%, respectively. Anemic pregnant women with a low household income had the highest prevalence rates of double (32.5%) and triple (15.8%) erythropoiesis-related micronutrient deficiencies. Dietary pattern scores were positively correlated with nuts and seeds, fresh fruits, total vegetables, breakfast cereals/oats and related products, soybean products, and dairy products but negatively correlated with processed meat products and liver, organs, and blood products. After adjusting for covariates, the dietary pattern had 29% (odds ratio (OR): 0.71; 95% confidence interval (CI): 0.055–0.091, *p* = 0.006)) and 43% (OR: 0.57; 95% CI: 0.41–0.80, *p* = 0.001)) reduced odds of having double and triple erythropoiesis-related micronutrient deficiencies for those pregnant women with a low household income. For those women with anemia, dietary patterns had 54% (OR: 046, 95% CI: 0.27–0.78) and 67% (OR: 0.33; 95% CI: 0.170.64) reduced odds of double and triple erythropoiesis-related micronutrient deficiencies. In conclusion, increased consumption of breakfast cereals and oats, nuts, and seeds, fresh fruits and vegetables, soybean products, and dairy products may protect women against erythropoiesis-related micronutrient deficiencies during pregnancy.

## 1. Introduction

Globally, anemia affects 56 million pregnant women in 2021 [1], especially women with a low household income level [2]. It was demonstrated that erythropoiesis triggers at a very early stage and half of the increase may occur within the first trimester using a direct measurement of red cell mass using a nonradioactive approach [3]. The erythropoietic system needs a constant supply of micronutrients (e.g., iron, vitamin (vit.) B_12_, and folic acid), and these demands further increase during pregnancy in order to support fetal growth and development. For example, erythroblasts need iron for hemoglobin (Hb) synthesis and folate and vit. B_12_ for proliferation during their differentiation [4].

In developing countries, prevalence rates of erythropoiesis-related micronutrient deficiencies among pregnant women in rural areas were 67.7%, 26.3%, and 74.1%, for iron, folate, and vit. B_12_, respectively [5]. Deficiencies in erythropoiesis-related micronutrients may lead to the development of gestational anemia [6], including iron deficiency (ID) anemia (IDA) or megaloblastic anemia [7]. An insufficient iron supply can result in microcytic hypochromic anemia. Megaloblastic anemia is caused by a deficiency of folic acid, vit. B_6_, and vit. B_12_. Deficiencies of those components of the vit. B complex lead to abnormal cell division and maturation manifested as a larger megaloblast, the precursors of red blood cells (RBCs) in the bone marrow, and enlarged (macrocytic) erythrocytes in the blood [8]. Importantly, deficiencies of iron, folate, and vit. B_12_ not only cause anemia but are also associated with several short- and long-term health risks for both the mother and child [9,10,11,12,13]. A low maternal folate status, especially with the concomitant presence of a vit. B_12_ deficiency, is recognized as a risk for neural tube defects, intrauterine growth retardation, preterm birth, and a low birth weight [9,10,11]. Gestational ID is associated with fetal growth restriction, offspring obesity, high blood pressure later in life [12], and impairment of neurodevelopment [13].

Insufficient dietary intake is the major cause of erythropoiesis-related micronutrient deficiencies [14]. The presence of micronutrient deficiencies during early gestation suggests that preexisting stores of micronutrients (e.g., iron and folate) were inadequate prior to pregnancy. Alternatively, the intake of micronutrients, either through dietary or supplemental sources, is insufficient to meet the demands of fetal development. Recommended dietary allowances (RDAs) of pregnant women for iron, folate, and vit. B_12_ are 27 mg/day, 600 μg/day, and 2.6 mg/day, respectively [15]. Pregnant women consuming vegetable- or plant-based diets are at increased risk of erythropoiesis-related micronutrient deficiencies [16]. Unlike other micronutrients, vit. B_12_ is mainly derived from meat products, and consuming a plant-based diet increases the risk of a vit. B_12_ deficiency. Meat also contains heme iron, which is more easily absorbed than nonheme iron [17]. Legumes, grains, nuts, seeds, fruits, and vegetables supply good amounts of iron, vit. B_6_, and folate [18]. However, they also contain phytate, which reduces nonheme iron bioavailability. The concomitant presence of vit. C increases nonheme iron bioavailability [18,19]. Nonetheless, use of prenatal supplements must be considered for the wellbeing of the fetus, especially for pregnant women whose dietary patterns are mainly vegetable- or plant-based [16].

Household income is a primary economic aspect that affects dietary preferences and the nutritional status of individuals [20]. Results from the National Health and Nutrition Examination Survey (NHANES) from 1999 to 2002 in the United States (US) showed that lower proportions of low-income US adults ate above or equal to the adequate intake (AI) or estimated average requirement (EAR) intake levels for many micronutrients [20]. Data from 18 publications originating primarily from western Europe showed a positive relationship between socioeconomic status and micronutrient status [21]. Specifically, women in resource-poor settings often had low micronutrient intake, such as folate, vit. B_12_, vit. C, vit. A, and Fe (below the EAR) [14,22]. Sociodemographic factors also determined dietary pattern adherence during pregnancy, for which a healthy diet was associated with household income [23]. A survey in China found that urban pregnant women had greater food diversity than did rural pregnant women [24]. Overall, those studies highlighted the importance of socioeconomic inequalities in diet-related diseases especially among pregnant women.

The use of a dietary pattern analysis to understand nutritional risk factors affecting pregnancy outcomes is becoming more popular [25]. Dietary pattern analyses take into account cumulative and interactive effects among dietary food groups to reflect the complexity of the human diet [26]. By studying nationwide representative population data in Taiwan, the broad aims of this study were to investigate (1) the prevalence of erythropoiesis-related micronutrient deficiencies among pregnant women stratified by household income and anemic status, and (2) the identification of dietary patterns with protective effects against erythropoiesis-related micronutrient deficiencies, especially for women with low household income and an anemic status.

## 2. Materials and Methods

### 2.1. Data Source and Participants

A population-scale cross-sectional survey, the Nationwide Nutrition and Health Survey in Taiwanese Pregnant Women (NAHSIT-PW), was conducted between 2017 and 2019. Details of the study design can be seen in previous publications [7,19]. Briefly, inclusion criteria were pregnant women aged ≥ 15 years, with Taiwanese residency who spoke fluent Chinese or Taiwanese, had received a maternal health checkout booklet, and for whom the legal guardian had a signed written informed agreement for those who were ≤19 years of age. For this analysis, we excluded participants who were ≤19 years of age (*n* = 6), who had nonsingleton pregnancies (*n* = 33), and who had missing information on household income (*n* = 32). In total, 1437 respondents were included in the current analysis.

### 2.2. Ethics

The study protocol was approved by the institutional review board of the Taipei Medical University (TMU-JIRB N201707039). Eligible participants provided written informed consent.

### 2.3. Data Collection

Baseline data on pregnant women were collected using a self-reported questionnaire including household income, residential area, anthropometrics, the use of prenatal supplements (such as multivitamins, vit. B_12_, folate, and iron), and disease history before and during the pregnancy. A face-to-face interview with an experienced dietitian was conducted to collect a 24 h dietary recall (24-HDR) and food frequency questionnaire (FFQ). The ratio of weight (in kg)-to-height squared (in m^2^) was used as the basis for the estimation of the pre-pregnancy body mass index (pBMI). To assess nutritional status, blood samples were taken during prenatal care.

### 2.4. Blood Biochemistry and Definition of Erythropoiesis-Related Micronutrient Deficiencies

Each participant underwent a phlebotomy in the hospital’s blood collection room. A complete blood cell count and Hb content in whole blood were measured (Hematology Analyzer, Sysmex, Kobe, Japan). Serum was used to determine contents of iron (e.g., iron, ferritin, and hepcidin), folate, and vit. B_12_. Serum ferritin (chemiluminescence immunoassay using a Beckman Coulter Unicel DxC 800 (Beckman Coulter, Brea, CA, USA)), total iron-binding capacity (TIBC) (immunoturbidimetric method by Beckman Coulter), and serum iron (ferrozine-based colorimetric assay using a Beckman Coulter Unicel DxC 800) were analyzed at Le Zen Clinical Laboratory (Taipei, Taiwan). Transferrin saturation (TS) was estimated as serum iron divided by the TIBC as a percentage (%). Hepcidin was measured using a Duo-Set enzyme-linked immunosorbent assay (ELISA) (R&D System, Minneapolis, MN, USA).

Trimesters (Trs) were defined as (1) Tr1, 17 weeks of gestation that follows the last normal menstrual cycle; (2) Tr2, weeks 18–28; and (3) Tr3, weeks 29–40 based on recommendations of the Ministry of Health and Welfare, Taiwan. According to the Center for Disease Control and Prevention (CDC), anemia was defined as Hb of <110 g/L in the first and third trimesters and Hb of <105 g/L in the second trimester. Following our previous study, ID was defined as TS of <16% without serum ferritin as biomarker to avoid ‘false-negative’ diagnoses among pregnant women experiencing chronic inflammation (such as obesity) [7]. Folate depletion was defined as serum folate of 6 ng/mL. A vit. B_12_ deficiency was defined as a serum vit. B_12_ level of <203 pg/mL [7]. Erythropoiesis-related micronutrient deficiencies were defined as (1) normal: none of the tested micronutrient deficiencies was present; and (2) single, double, and triple (multiple) micronutrient deficiencies: the presence of one, two, or three of the following: ID, folate depletion, or a vit. B_12_ deficiency.

### 2.5. Dietary Assessment

Dietary intake was assessed using a 24-HDR. Briefly, participants were asked to recall detailed information about all food and beverages they had consumed in the past 24 h. Detailed information (e.g., meal type, meal time, food sources, items consumed, and cooking techniques) was also collected to help determine the daily intake of nutrient levels. All dietary data were standardized based on the Taiwan Food Nutrient Database, using the internet-based program Cofit Pro (Cofit Healthcare, Taipei, Taiwan). An interviewer-administered FFQ was conducted, and FFQ data were used to generate dietary pattern scores. Respondents were asked how often (daily, weekly, and monthly) they had consumed each food item in the previous month. The NAHSIT-PW 2017–2019 uses a 59-item FFQ to appraise the dietary status of the preceding 1-month period. The detailed FFQ is described elsewhere [19]. To generate dietary pattern scores, 59 food items were merged into 33 food groups according to the similarity of food characteristics and nutritional components.

### 2.6. Statistical Analysis

We calculated the mean ± standard deviation (SD) for continuous data and number (percentage) for categorical data. General linear models for continuous data and χ-squared test for categorical data were employed to generate *p* for trend values. We subsequently applied a reduced rank regression (RRR) using the PROC PLS function in SAS 9.4 (SAS, Cary, NC, USA) to derive dietary patterns predictive of erythropoiesis-related micronutrient deficiencies. Selected nutrient intakes were adjusted using the nutrient density model [27] and not normally distributed response variables were logarithmically transformed before the RRR procedure. Briefly, the RRR identified linear functions of predictors (e.g., 33 food groups) that explained as much of the response variation as possible (e.g., folate, household income levels, protein (%), vit. C, and TS (%)). We only selected the first factor which accounted for the highest variation among the response variables. Dietary pattern scores were categorized into tertiles (Ts), with the highest group (T3) conforming most closely to the identified dietary pattern. Participants’ characteristics were summarized according to the T of the dietary pattern score. The odds of erythropoiesis-related micronutrient deficiencies were examined using a multinomial logistic regression, which generated adjusted odds ratios (ORs) and 95% confidence intervals (CIs) for the risk of erythropoiesis-related micronutrient deficiencies. *p* for trend was conducted using generalized linear models. Data were analyzed using GraphPad Prism 5 (GraphPad Prism 5, GraphPad Software, San Diego, CA, USA) and SPSS ver. 21 (IBM, Armonk, NY, USA) with *p* for trend and *p*-value < 0.05 considered significant.

## 3. Results

### 3.1. Relationships of Anemia and Household Income with Erythropoiesis-Related Micronutrient Deficiencies

**Figure 1:**
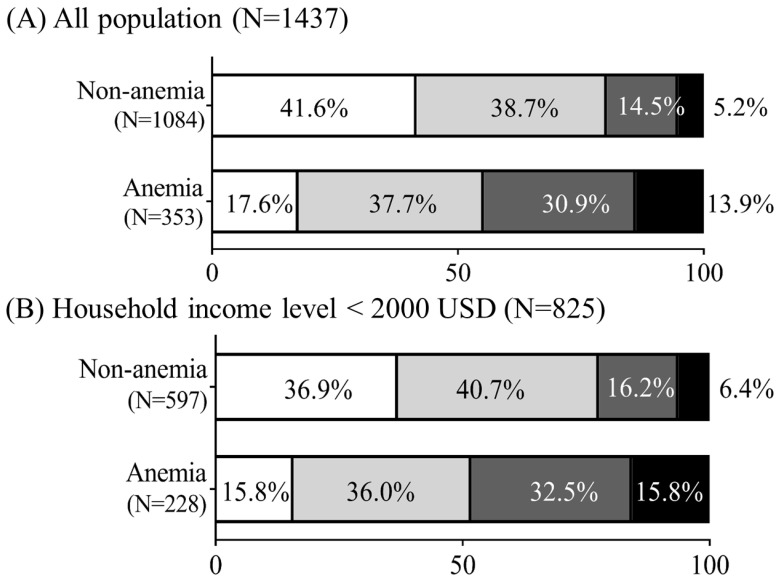

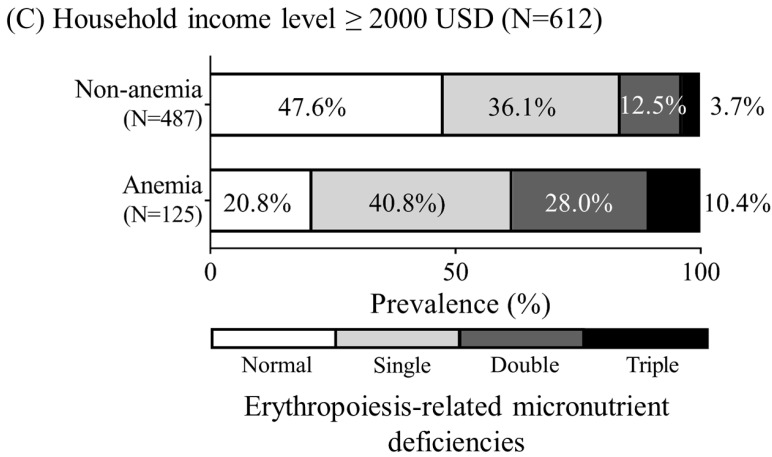
Prevalences of erythropoiesis-related micronutrient deficiencies in relation to household income and the anemic status among pregnant women. (**A**) The total population. (**B**) Household monthly income levels of ≥USD2000. (**C**) Household monthly income levels of <USD2000. Definitions: erythropoiesis-related micronutrient deficiencies: (1) normal: none of the tested micronutrient deficiencies were present; (2) single (one), double (two), and triple (three) micronutrient deficiencies: the presence of one, two, or three of the following: iron deficiency (ID), folate depletion or a vitamin (vit.) B_12_ deficiency. Anemia: hemoglobin (Hb) < 11 g/dL (in the first and third trimesters), and Hb < 10.5 g/dL (in the second trimester); ID: transferrin saturation (TS) < 15%; folate depletion: serum folate < 6 ng/mL; vit. B_12_ deficiency: serum vit. B_12_ < 203 pg/mL.

Overall prevalences of normal status, and single, double, and triple erythropoiesis-related micronutrient deficiencies were 35.7%, 38.2%, 18.6%, and 7.5%, respectively. Figure 1A shows that compared to nonanemic pregnant women, anemic pregnant women had higher rates of double (30.9% vs.14.5%) and triple (13.9% vs. 5.2%) erythropoiesis-related micronutrient deficiencies. We next stratified women according to anemia and household income levels (high (≥USD2000) and low (<USD2000). Among nonanemic pregnant women, women with a high household income had the highest prevalence rate of a normal status (47.6% vs. 36.9%) but the lowest rates of double (12.5% vs. 16.2%) and triple erythropoiesis-related micronutrient deficiencies (3.7% vs. 6.4%) compared with those with a low household income (Figure 1B,C). Among anemic pregnant women, women with a low household income had the highest prevalence rates of double (32.5% vs. 28%) and triple (15.8% vs. 10.4%) erythropoiesis-related micronutrient deficiencies compared to those with a high household income level (Figure 1B,C).

### 3.2. Erythropoiesis-Associated Dietary Pattern Scores Using the RRR

Erythropoiesis-related dietary pattern scores were derived using the RRR from 33 food groups. Response variables were household income, serum folate, %TS, dietary vit. C, and % protein intake (Supplementary Table S1), which were selected based on a literature review.

Table 1 shows the first dietary pattern scores, of which response variables explained 15.17% of the variation. Dietary pattern scores were positively correlated with nuts and seeds, fresh fruits, total vegetables, breakfast cereals/oats and related products, soybean products, and dairy products (factor loadings of ≥0.20) but negatively correlated with processed meat products and liver, organs, and blood products (factor loadings of ≤−0.20).

### 3.3. Maternal Characteristics Stratified by Erythropoiesis-Associated Dietary Pattern Scores

Table 2 shows that pregnant women with the lowest dietary pattern scores (T1) were younger, heavier, and had the highest rates of less than an undergraduate degree (23.9%) and low household incomes (69.5%) (all *p* for trend < 0.05). Dietary pattern scores were negatively correlated with the prevalence of erythropoiesis-related micronutrient deficiencies but positively correlated with blood biomarkers (serum iron, %TS, vit. B_12_, and folic acid) (all *p* for trend < 0.05). Significant positive linear trends were also observed among dietary pattern scores, self-reported prenatal dietary supplements used (total supplement and multivitamin/minerals), and adjusted nutrient intake levels (percentage of protein intake, fiber intake, total sugar, folate, dietary vit. B_12_, vit. C, and vit. A) (all *p* for trend < 0.05).

### 3.4. Protective Effects of Dietary Patterns on Erythropoiesis-Related Micronutrient Deficiencies in Relation to Household Income and Anemic Status

**Figure 2:**
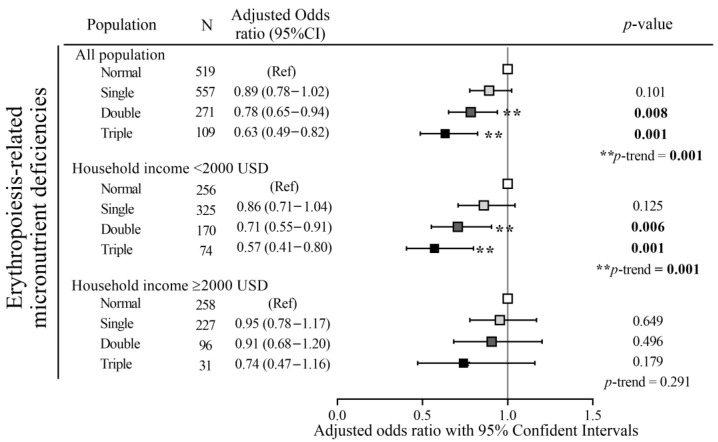
Adjusted odds ratios (ORs) and 95% of confidence intervals (CI) of erythropoiesis-related micronutrient deficiencies with dietary pattern scores as a predictor stratified by household income. ORs were calculated using a multinomial logistic regression. *p* for trend was tested using a generalized linear model, which was adjusted for age, pre-pregnancy body mass index, region, trimester, parity, (%) total supplement, and calorie intake. In 2019, the average exchange rate was USD1.0 ≈ TWD30. Between 2017 and 2019, the average monthly household income per capita was TWD13, 0.855–14.33 ≈ USD1155–1194. *p* value/*p* for trend as ** *p* ≤ 0.01.

We next performed a multinomial logistic regression analysis to investigate the protective effect of dietary patterns on the risk of erythropoiesis-related micronutrient deficiencies. After adjusting for covariates (age, pBMI, region, trimester, parity, percentage of total dietary supplement, and calorie intake), dietary pattern scores showed 22% (OR: 0.78; 95% CI: 0.65–0.94, *p* = 0.008) and 37% (OR: 0.63; 95% CI: 0.49–0.82, *p* = 0.001) reduced odds of having double and triple erythropoiesis-related nutritional deficiencies, respectively (Figure 2, all populations). Among pregnant women with low household income, dietary pattern scores showed 29% (OR: 0.71; 95% CI: 0.055–0.091, *p* = 0.006)) and 43% (OR: 0.57; 95% CI: 0.41–0.80, *p* = 0.001)) reduced odds of having double and triple erythropoiesis-related nutritional deficiencies after adjusting for covariates (age, pBMI, region, trimester, parity, percentage of total dietary supplement, and calorie intake) (Figure 2). No significant protective effects of dietary patterns were detected for pregnant women with a high household income after adjusting for covariates.

**Figure 3:**
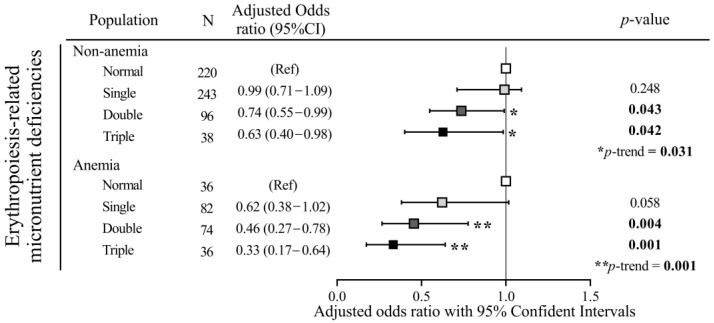
Adjusted odds ratios (ORs) and 95% confidence intervals (CIs) of erythropoiesis-related micronutrient deficiencies with dietary pattern scores as a predictor stratified by anemia. ORs were calculated using a multinomial logistic regression. *p* for trend was tested by a generalized linear model, which was adjusted for age, region, trimester, and total supplement use (%). Anemia was defined as hemoglobin (Hb) levels of <11 g/dL (in the first and second trimesters), and Hb levels of <10.5 g/dL (in the second trimester). *p* value/*p* for trend as * *p* ≤ 0.05, ** *p* ≤ 0.01.

Figure 3 shows that for those pregnant women with anemia, erythropoiesis-related dietary patterns had 54% (OR: 046, 95% CI: 0.27–0.78), and 67% (OR: 0.33; 95% CI: 0.17–0.64) reduced odds of developing double and triple erythropoiesis-related micronutrient deficiencies, respectively (all *p* < 0.01). Dietary pattern scores also protected nonanemic pregnant women against double (OR: 0.74; 95% CI: 0.055–0.099, *p* = 0.043) and triple (OR: 0.63; 95% CI: 0.40–0.98, *p* = 0.042) erythropoiesis-related micronutrient deficiencies.

## 4. Discussion

By studying the micronutrient status of a nationwide representative 1430 pregnant women, the present study shows that the prevalence of erythropoiesis-related micronutrient deficiency was greater among pregnant women with anemia or low household income compared to those who did not have those features. We identified erythropoiesis-related dietary patterns, mainly plant-based foods (nuts and seeds, fresh fruits, total vegetables, breakfast cereals/oats and related products, soybean products, and dairy products, and a low intake frequency of processed meat and blood/liver/organs and related products) that can supply adequate micronutrients for erythropoiesis in the bone marrow and reduced the risk of micronutrient deficiencies during pregnancy. Importantly, the protective effect of the dietary pattern was higher for pregnant women with low household income and anemia.

It is well established that a constant supply of micronutrients such as iron, folate, and vit. B_12_ are essential for erythropoiesis, and inadequate supplies of any of these may increase the risk of gestational anemia [8]. In many developing countries, prevalence rates of anemia are up to 75%, and micronutrient deficiencies, especially iron deficiency, are believed to be the main underlying cause of anemia [6]. The current study results agree with findings from the US National Health and Nutritional Survey [28]. The current study found that among pregnant women, prevalences of single (38.2%), double (18.6%), and triple micronutrient deficiencies (7.5%) were similar to those of US pregnant or breastfeeding women, of which the prevalences of one, two, and multiple (≥3–5) micronutrient deficiencies were 30%, 13%, and 1.5%, respectively [28]. However, the prevalence rate of multiple (triple) erythropoiesis-related micronutrient deficiencies among Taiwanese pregnant women (7.5%) was lower than that of Indian pregnant women living in a rural area (16.2%) [5]. In agreement with a community based-study in India [5], anemic pregnant women (82.4%) had a higher rate of developing micronutrient deficiencies than nonanemic pregnant women (58.4%).

The identified erythropoiesis-related micronutrient dietary pattern scores protected the women against the development of double and triple erythropoiesis-related micronutrient deficiencies (*p* < 0.05). Specifically, a one-unit increase in the dietary pattern score was respectively associated with 22% and 37% reduced risks of having double and triple erythropoiesis-related micronutrient deficiencies during pregnancy. This suggests that pregnant women should be encouraged to consume plant-based foods (e.g., nuts and seeds, fresh fruits, vegetables, breakfast cereals/oats, and soybean products) and dairy products but should decrease the intake frequency of processed meats and blood/liver/organ-related products. In general, erythropoiesis-related micronutrient dietary patterns were similar to recommendations of a healthy diet for pregnant women. Pregnant women are encouraged to eat a variety of nutrient-dense foods from plant products as these natural food products are rich in iron minerals and vitamins and also protein and α-linoleic acid compared to those highly processed meat products [29].

Erythropoiesis-related micronutrient dietary pattern scores accounted for 104.13% of the total explained variation, with nuts and seeds the strongest at 29.33%, followed by fresh fruits (23.22 %), vegetables (12.64%), and breakfast cereal and oat-related products at 9.50% (Table 1). Nuts and seeds were positively correlated with all response variables, except for %TS (all *p* < 0.05) (Supplementary Table S2). Fresh fruits were positively associated with serum folate (ß = 0.022) and vit. C intake frequency (ß = 0.922) but negatively correlated with %TS (ß = −0.027) (Supplementary Table S2). A previous cluster study [18] showed that nuts and seeds, fruits, vegetables, and cereal grains contain iron (minimum (min)–maximum (max): 1.64–14.55, 0–1.02, 0.06–8.9, and 0.8–7.61 mg) and folate (min–max: 11–22, 0–89, 3–338, and 7–281 μg), vit. C (min–max: 0–46.6, 0.4–228, 0–242.5, 0–4.2 mg) per 100 g, respectively. A study among 725 older men and 705 older women from the Nutrition and Health Survey in Taiwan (1999–2000) (Elderly-NASHIT) found that vegetables (66%) and fruits (11.8%) were the main sources of folate [30]. This suggests that a higher folate intake is achievable due to the abundance of a diverse variety of fruit and vegetables in Taiwan [30]. Dairy products accounted for 6.57% of the total explained variation (Table 1), and this food group was significantly correlated with response variables, except for %TS (Supplementary Table S2). Gille and Schimd showed that dairy products and meats are sources of vit. B_12_ [31]. Processed meat products explained 10.57% of the total explained variation (Table 1), but they were significantly negatively correlated with %TS, serum folate, and household income levels (Supplementary Table S2). The Biomarker Reflecting Inflammation and Nutritional Determinant of Anemia (BRINDA) project revealed that higher red and processed meat consumption was positively associated with increased levels of proinflammatory markers among overweight and obese women [32]. Adiposity-mediated low-grade inflammation is known to reduce iron bioavailability due to increased hepcidin levels that block iron absorption and iron efflux [33,34].

The present study found that dietary vit. C contributed 3.98% to the total explained variation of response variables (Supplementary Table S1). Vitamin C intake is known to influence blood iron status. Vitamin C is an iron enhancer agent that can improve the nonheme iron bioavailability of plant-based foods such as dark leafy vegetables, fruits, and cereals and protects against IDA [19]. We found a strong positive trend between vit. C intake and dietary pattern scores (*p* < 0.001). We observed that one unit increase in fresh fruit frequency significantly increased 0.922 mg intake of vit. C (*p* < 0.001) (Supplementary Table S2). Abdul-Fattah and colleagues conducted a cross-sectional study among pregnant women and found that poor dietary intake of vegetables, fresh fruits, and dry fruits was associated with IDA [35].

Our study found that household income levels were positively associated with dietary pattern scores (ß = 0.335, 95% CI: 0.214–0.455, *p* < 0.001). Specifically, we found that dietary pattern scores protected against double (OR: 0.71; 95% CI: 0.55–0.91, *p* = 0.006) and triple (OR: 0.57; 95% CI: 0.41–0.80, *p* = 0.001) erythropoiesis-related micronutrient deficiencies among pregnant women with low household income but not high household income. This is in agreement with De Castro and colleagues, who conducted a cross-sectional study among pregnant women and found that monthly per capita family income was a factor related to adherence to a healthy dietary pattern (mostly consisting of legumes, vegetables, and fruits) [23]. This suggests that women with a high household income level (≥USD2000) would more likely adhere to a healthy dietary pattern than those with a low household income level, and this may protect them against micronutrient deficiencies during pregnancy. Bowman et al. also found a positive relationship between income and micronutrients intake [20]. In low-income level settings, a possible solution is to increase dietary diversity [24], especially by diversifying planned food consumption [18]. The positive correlation observed between micronutrient consumption and socioeconomic status in our study highlights the importance of socioeconomic disparities in diet-related diseases among disadvantaged populations [21].

Another important finding of our study was the protective effect of the dietary pattern among anemic and nonanemic pregnant women. The present study found that, among pregnant women with anemia, dietary pattern scores showed 54% and 67% reduced risks of double (OR: 0.46, 95% CI: 0.27–0.78, *p* = 0.004) and triple (OR: 0.33, 95% CI: 0.17–0.064, *p* = 0.001) erythropoiesis-related micronutrient deficiencies, while among nonanemic pregnant women, dietary pattern scores showed 26% and 37% reduced risks for double (OR: 0.74, 95% CI: 0.55–0.99, *p* = 0.043) and triple (OR: 0.63, 95% CI: 0.40–0.98, *p* = 0.042) erythropoiesis-related micronutrient deficiencies, respectively. A previous study also suggested that women who did not maintain World Health Organization-recommended levels of adequate fruit and vegetable intake (five or more servings/day) had an increased risk of being moderately to severely anemic [36]. Paramastri et al. [37] and Kurniawan et al. [38] identified an anemia-inflammatory dietary pattern, characterized by high intake frequencies of eggs, meat, processed foods, and sugary beverages, but lower intake of vegetables and fruits. Taken together, those studies emphasize the importance of the consumption of a diversity of plant-based foods during pregnancy.

Our current study includes a large sample of pregnant women representing the Taiwanese population, which allowed us to have more precise and robust estimates of the relationship between dietary patterns and erythropoiesis-related micronutrient deficiencies. However, our study also had several limitations that need to be taken into account when translating the findings. First, our cross-sectional study design was unable to address causality. Second, we included only single 24 h recall data, which might not eliminate recall error. Third, our study relied on self-administered dietary data (e.g., an FFQ), for which systematic underreporting or recall bias of energy intake may have been present. It was discovered that pregnant women overreported their food intake when using an FFQ as a dietary survey tool [39]. Nonetheless, in large-scale epidemiological studies, the FFQ is regarded as one of the most appropriate methods for dietary assessment, and it presents useful information on dietary patterns [40]. Our study used transferrin saturation without ferritin to define ID, which may affect the accuracy of the diagnosis. A previous study suggested that transferrin saturation has 61% sensitivity and 86% specificity compared to serum ferritin or bone marrow examination as the gold standard [41]. However, our definition may also reduce false negative diagnoses of ID for those who are elevated ferritin concentration because of chronic inflammation.

## 5. Conclusions

In conclusion, the erythropoiesis-associated dietary pattern, mostly plant-based foods, characterized by high intake frequencies of nuts and seeds, fresh fruits, total vegetables, breakfast cereals/oats and related products, soybean products, and dairy products, may prevent the development of micronutrient deficiencies during pregnancy, especially for women with low household income or an anemic status.

## Figures and Tables

**Table 1:** Food groups associated with the first factor of erythropoiesis-related dietary pattern scores identified using a reduced rank regression (RRR).

	Explained Variation (%)	Factor Loading *
Food groups		
Positive association		
- Nut and seeds	29.33	0.48
- Fresh fruits	23.22	0.43
- Total vegetables	12.64	0.31
- Breakfast cereals/oats and related products	9.50	0.27
- Soybean products	7.28	0.24
- Dairy products	6.57	0.23
Negative association		
- Processed meat products	10.57	−0.29
- Liver, organs, and blood products	5.02	−0.20
Total explained variation	104.13	

* Factor loadings are correlations between food groups and dietary pattern scores (correlation coefficient for the RRR-derived pattern ≥ |0.20|).

**Table 2:** Characteristic of study participants according to tertile (T) groups of erythropoiesis-related dietary pattern scores.

Variable	Dietary Pattern Scores ^#^			*p* for Trend
	T1 (*N* = 479)	T2 (*N* = 479)	T3 (*N* = 479)	
Basic characteristic				
Age (years)	31.63 ± 4.93	32.54 ± 4.56	33.51 ± 4.38	<0.001
pBMI (kg/m^2^)	22.87 ± 4.38	22.78 ± 4.14	22.25 ± 3.35	0.016
Underweight (*n*, %)	51 (10.7)	44 (9.2)	45 (9.4)	0.129
Normal weight (*n*, %)	282 (59.2)	284 (59.5)	312 (65.3)	
Overweight (*n*, %)	73 (15.3)	77 (16.1)	73 (15.3)	
Obesity (*n*, %)	70 (14.7)	72 (15.1)	48 (10.0)	
Trimester				
First (*n*, %)	129 (26.9)	118 (24.6)	111 (23.2)	0.198
Second (*n*, %)	151 (31.5)	157 (32.8)	155 (32.4)	
Third (*n*, %)	199 (41.5)	204 (42.6)	213 (44.5)	
Educational level				
Less than undergraduate (*n*, %)	114 (23.9)	67 (14.0)	35 (7.3)	<0.001
Undergraduate or above (*n*, %)	362 (76.1)	410 (86.0)	443 (92.7)	
Household income level (USD)				
<2000 (*n*, %)	333 (69.5)	260 (54.3)	232 (48.4)	<0.001
≥2000 (*n*, %)	146 (30.5)	219 (45.7)	247 (51.6)	
Blood biomarkers				
Serum hepcidin (ng/mL)	23.03 ± 32.56	24.84 ± 32.43	24.21 ± 32.14	0.573
Serum iron (µg/dL)	67.23 ± 37.13	75.08 ± 43.06	74.37 ± 39.37	0.006
Log TS (%)	15.48 ± 9.61	17.39 ± 10.38	16.84 ± 9.87	0.034
Hemoglobin (g/dL)	11.69 ± 2.01	11.82 ± 2.07	11.72 ± 1.74	0.813
Serum ferritin (ng/mL)	22.92 ± 27.99	24.86 ± 26.32	21.96 ± 22.07	0.564
Folic acid (ng/mL)	10.92 ± 6.84	13.07 ± 7.42	14.19 ± 6.98	<0.001
Log vit. B_12_ (pg/mL)	309.59 ± 242.63	316.39 ± 207.02	316.95 ± 138.02	0.031
Erythropoiesis-related nutritional deficiencies				
Normal (*n*, %)	139 (29.0)	174 (36.3)	201 (42.0)	<0.001
Single (*n*, %)	178 (37.2)	198 (41.3)	176 (36.7)	
Double (*n*, %)	103 (21.5)	78 (16.3)	85 (17.7)	
Triple (*n*, %)	59 (12.3)	29 (6.1)	17 (3.5)	
Reported use of prenatal dietary supplements				
Total supplement use (*n*, %)	364 (77.4)	409 (86.1)	427 (90.1)	<0.001
Multivitamin/mineral (*n*, %)	250 (53.2)	311 (65.8)	325 (69.1)	<0.001
Vit. B (*n*, %)	88 (18.8)	84 (18.1)	87 (18.4)	0.872
Folate (*n*, %)	217 (46.0)	221 (47.3)	214 (45.6)	0.860
Iron (*n*, %)	46 (9.8)	42 (9.0)	63 (13.4)	0.073
Adjusted nutrients intake status				
Carbohydrates (g/day)	124.15 ± 25.14	124.67 ± 24.56	126.29 ± 23.28	0.174
Protein (g/day)	37.62 ± 9.61	38.10 ± 8.88	39.03 ± 9.47	0.019
Fat (g/day)	40.27 ± 9.98	40.01 ± 10.21	39.09 ± 9.49	0.065
Log dietary Fe (mg/day)	5.75 ± 4.54	5.99 ± 3.83	6.02 ± 2.60	0.001
Log dietary folate (mg/day)	113.27 ± 69.05	117.65 ± 53.71	119.77 ± 48.03	0.006
Log dietary vit. B_12_ (µg/day)	2.80 ± 5.12	2.99 ± 4.49	3.41 ± 7.02	0.02
Log dietary vit. C (mg/day)	54.00 ± 66.04	63.56 ± 67.75	78.09 ± 83.41	<0.001
Log dietary vit. A (µg RE/day)	404.17 ± 805.96	436.52 ± 692.95	508.50 ± 577.59	<0.001
Under RDA of protein (*n*, %)	195 (41.0)	199 (41.6)	158 (33.0)	0.011
Under RDA of Fe (*n*, %)	441 (92.6)	433 (90.6)	440 (91.9)	0.660
Under RDA of folate (*n*, %)	459 (96.4)	457 (95.6)	459 (95.8)	0.637
Under RDA of vit. B12 (*n*, %)	231 (48.5)	209 (43.7)	186 (38.8)	0.003
Under RDA of vit. C (*n*, %)	356 (74.8)	317 (66.3)	252 (52.6)	<0.001

Continuous data are represented as the mean ± standard deviation, whereas categorical data are represented as the number (percentage of the same group). *p* for trend was tested using a generalized linear model/one-way ANOVA for continuous data and chi-squared for categorical data. ^#^ Dietary pattern scores in tertiles (Ts): Tertile 1 (T1) = −5.55–0.41; Tertile 2 (T2) = −0.40–0.27; Tertile 3 (T3) = 0.28–5.62. *p* for trend was tested using a general linear model/one-way ANOVA for continuous data and chi-squared for categorical data. Abbreviations: pBMI, pre-pregnancy body-mass index; TS: transferrin saturation; vit.: vitamin; RDA: recommended daily amount. In 2019, the average exchange rate was 1.0 USD ≈ 30 New Taiwanese Dollars (TWD). Between 2017 and 2019, the average monthly household income per capita was TWD13,855–14,332 ≈ USD1155–1194. Definitions: erythropoiesis-related micronutrient deficiencies: (1) normal: no nutritional deficiency was present; (2) single (one), double (two), and triple (three) micronutrient deficiencies: the presence of one, two, or three iron deficiencies (ID); folate depletion; or a Vit. B_12_ deficiency. ID: TS < 15%, folate depletion: serum folate < 6 ng/mL; Vit. B_12_ deficiency: serum Vit. B_12_ < 203 pg/mL.

## Data Availability

Upon reasonable request, the corresponding author will provide data described in the manuscript, code book, and analytic code.

## Supplementary Materials

The following supporting information can be downloaded at: https://www.mdpi.com/article/10.3390/nu15102311/s1, Table S1: Response variables identified by the reduced rank regression; Table S2: Beta (β) coefficients and 95% confidence intervals (CIs) of response variables for individual for food groups of erythropoiesis-related dietary pattern scores.

## Abbreviations

vit.: vitamin; Hb: hemoglobin; ID: iron deficiency; IDA: iron deficiency anemia; RBCs: red blood cells; NHANES: the National Health and Nutrition Examination Survey; US: the United States; AI: adequate intake; EAR: estimated average requirement; NAHSIT-PW: the Nationwide Nutrition and Health Survey in Taiwanese Pregnant Women; 24-HRD: 24-h dietary recall; FFQ: food frequency questionnaire; pBMI: pre-pregnancy body mass index; TIBC: total iron-binding capacity; TS: transferrin saturation; ELISA: enzyme-linked immunosorbent assay; Trs: trimesters; CDC: Center for Disease Control and Prevention; SD: standard deviation; RRR: reduced rank regression; Ts: tertiles; ORs: odds ratios; CIs: confidence intervals; NAHSIT: the Nutrition and Health Survey in Taiwan; BRINDA: the biomarker reflecting inflammation and nutritional determinant of anemia.
