# Supplementary material for: Relationships between Dietary Patterns and Erythropoiesis-Associated Micronutrient Deficiencies (Iron, Folate, and Vitamin B12) among Pregnant Women in Taiwan
Source: Nutrients. 2023 May 15;15(10):2311. doi: 10.3390/nu15102311 (PMC10224368; doi:10.3390/nu15102311)
Supplement: Supplementary file 1 [file nutrients-15-02311-s001.zip › nutrients-2294874-supplementary.pdf]

Supplementary Table S1. Response variables identified by the reduced rank regression

	Explained variation (%)
Response (%)	
- Folate	4.80
- Household income levels	4.69
- log Vitamin C	3.98
- Protein (%)	1.02
- log TS	0.67
Total explained variation	15.17

TS, transferrin saturation.

Supplementary Table S2. Beta (ß) coefficient**s** and 95% confiden**ce** intervals (CI**s**) of response variables for individual for food group**s** of erythropoiesis-related dietary pattern scores

	Serum folate		Income level		Dietary **v**itamin C		Dietary **p**rotein (%)		TS (%)	
	ß (95% CI)	*p* value	ß (95% CI)	*p* value	ß (95% CI)	*p* value	ß (95% CI)	*p* value	ß (95% CI)	*p* value
Selected food groups [#]										
Breakfast cereals, oats**,** and related products	0.034 (-0.001~0.069)	0.058	0.019 (0.005~0.033)	0.008	0.667 (0.018~1.317)	0.044	0.019 (-0.001~0.040)	0.065	-0.025 (-0.076~0.026)	0.34
Dairy products	0.01 (-0.004~0.025)	0.133	0.004 (0.000~0.009)	0.074	0.192 (-0.068~0.452)	0.148	0.006 (-0.002~0.015)	0.127	-0.004 (-0.024~0.017)	0.74
Total vegetables	0.005 (-0.005~0.015)	0.323	0.005 (0.001~0.008)	0.008	0.191 (0.001~0.380)	0.048	0.008 (0.002~0.014)	0.011	-0.012 (-0.026~0.003)	0.13
Soybean products	0.028 (0.003~0.053)	0.029	0 (-0.008~0.009)	0.949	0.284 (-0.184~0.752)	0.234	-0.013 (-0.028~0.002)	0.087	-0.005 (-0.041~0.032)	0.805
Nut and seeds	0.049 (0.013~0.085)	0.008	0.013 (0.001~0.025)	0.033	0.819 (0.149~1.490)	0.017	0.025 (0.004~0.046)	0.021	-0.006 (-0.058~0.047)	0.833
Fresh fruits	0.022 (0.006~0.037)	0.005	0.004 (-0.001~0.009)	0.105	0.922 (0.639~1.205)	<0.001	0 (-0.009~0.009)	0.977	-0.027 (-0.049~-0.005)	0.018
Liver, organs and blood products	0.001 (-0.041~0.043)	0.946	-0.012 (-0.028~0.004)	0.155	-0.577 (-1.343~0.189)	0.14	-0.004 (-0.028~0.021)	0.776	0.024 (-0.037~0.085)	0.437
Processed meat products	-0.042 (-0.078~-0.006)	0.023	-0.014 (-0.027~-0.001)	0.04	-0.061 (-0.729~0.607)	0.858	-0.005 (-0.026~0.016)	0.629	-0.055 (-0.107~-0.002)	0.041
Dietary pattern scores#	0.984 (0.636~1.332)	<0.001	0.335 (0.214~0.455)	<0.001	14.222 (7.844~20.601)	<0.001	0.3 (0.095~0.505)	0.004	0.142 (-0.371~0.655)	0.587

[#] Adjusted for age, pre-pregnancy body-mass index (pBMI), trimester, parity, **and** total supplement use (%). Abbreviation: TS, transferrin saturation**.**
